# Introducing *Diagnostic Pathology*

**DOI:** 10.1186/1746-1596-1-1

**Published:** 2006-03-31

**Authors:** Klaus Kayser

**Affiliations:** 1 Director of the UICC-TPCC, Humboldt University (Charite), Institute of Pathology, Berlin, Germany

Welcome to the new journal ***Diagnostic Pathology ***[1]! This journal will be an open access, online journal encompassing all aspects of surgical pathology, including classic diagnostic pathology, prognosis-related diagnosis and therapy-related findings. There will be a focus on the technological aspects of pathology, including molecular biology techniques, morphometry aspects, communication aspects, electronic education, and quality assurance. Our primary aim, however, is to close the gap between these different specialisations within the field of diagnostic pathology. For this reason we also welcome articles from related fields in medicine and biology.

Why a new pathology journal, and why in a solely electronic presentation?

One reason to start ***Diagnostic Pathology ***follows from the statement: We want to publish submitted articles independent of any restrictions arising from the conditions imposed by regular print issues. Traditional print journals need to adjust the number of published articles to the available space. If space is a scarce resource, this may mean rejecting submissions that should be published or result in a long waiting time until publication. Alternatively, if the number of submissions is low, the situation may result in acceptance of articles that do not meet stringent quality criteria. These outcomes are undesirable and amount to non-scientific behavior. As an online journal that publishes articles immediately on acceptance, with no dictates from deadlines, issues and volumes, ***Diagnostic Pathology ***is under no pressure to publish any article that does not stand up to fair but strict peer review.

Our second motivation for launching is a response to another basically unscientific practice that colleagues loathe but often find forced to pursue. I am referring to the common practice of submitting to the journal with the highest available impact factor; if the article does not get accepted, the journal with the next lower ranking will be selected, and so on. What gets entirely ignored is the very point of conducting science: the dissemination of knowledge to anyone who is interested, be it as fellow researchers, patients, or in any other capacity. The practice described above more often than not leads to publication in journals that are inaccessible for many, if not the vast majority of people who would like to have access.

Traditional journals offer restricted access to articles. Often only the title, keywords, and abstract are available for free. The download of the full article then requires a payment by the requesting scientist or her/his institution. As a consequence, many researchers read only the abstract of the article they are interested in, rather than the full article. This practice is even worse as several journals restrict the abstract size of their articles to less than 300 words. From the scientific point of view, a far better strategy is to offer all readers unrestricted access to the full articles.

***Diagnostic Pathology ***will accept any publication as long as it is sound science and holds the potential for scientific impact – which, with the exception of a few rare discoveries, is largely unpredictable anyway. We are convinced that a very considerable number of research articles have been rejected by 'high score' journals due to various non-scientific reasons. The embedding of ***Diagnostic Pathology ***into the World Wide Web and its free global accessibility offers all published articles the judgment of the interested scientific community. Thus, the scientific value of an article published in ***Diagnostic Pathology ***is subject to the evaluation of the scientific community only.

To enable the journal to make all of its content open access, ***Diagnostic Pathology ***will levy an article-processing charge for each manuscript accepted after peer review (payable on acceptance). However, there will be an initial period without charges, and many colleagues will find that their institutions or funding agencies will cover the costs for them [2,3].

I can only encourage my colleagues to carefully weigh the scientific and financial arguments and to submit articles to ***Diagnostic Pathology ***as a new open access journal. We will certainly do our best to ensure that both sides, authors and the research community, will benefit from this new and exciting scientific initiative.
